# A high-resolution geospatial surveillance-response system for malaria elimination in Solomon Islands and Vanuatu

**DOI:** 10.1186/1475-2875-12-108

**Published:** 2013-03-21

**Authors:** Gerard C Kelly, Erick Hale, Wesley Donald, Willie Batarii, Hugo Bugoro, Johnny Nausien, John Smale, Kevin Palmer, Albino Bobogare, George Taleo, Andrew Vallely, Marcel Tanner, Lasse S Vestergaard, Archie CA Clements

**Affiliations:** 1 University of Queensland, Infectious Disease Epidemiology Unit, School of Population Health, Brisbane, Australia; 2 Ministry of Health and Medical Services, Vector Borne Diseases Control Programme, Honiara, Solomon Islands; 3 Ministry of Health, Vector Borne Diseases Control Programme, Port Vila, Vanuatu; 4 Griffith University, School of Public Health, Brisbane, Australia; 5 University of Hawaii, John A. Burns School of Medicine, Honolulu, USA; 6 University of New South Wales, The Kirby Institute for infection and immunity in society, Sydney, Australia; 7 Swiss Tropical & Public Health Institute, Basel, Switzerland; 8 University of Basel, Basel, Switzerland; 9 World Health Organization, Western Pacific Region, Manila, Philippines

**Keywords:** Malaria elimination, Surveillance, Geographic information systems (GIS), Spatial decision support systems (SDSS), Mapping

## Abstract

**Background:**

A high-resolution surveillance-response system has been developed within a geographic information system (GIS) to support malaria elimination in the Pacific. This paper examines the application of a GIS-based spatial decision support system (SDSS) to automatically locate and map the distribution of confirmed malaria cases, rapidly classify active transmission foci, and guide targeted responses in elimination zones.

**Methods:**

Customized SDSS-based surveillance-response systems were developed in the three elimination provinces of Isabel and Temotu, Solomon Islands and Tafea, Vanuatu. Confirmed malaria cases were reported to provincial malaria offices upon diagnosis and updated into the respective SDSS as part of routine operations throughout 2011. Cases were automatically mapped by household within the SDSS using existing geographical reconnaissance (GR) data. GIS queries were integrated into the SDSS-framework to automatically classify and map transmission foci based on the spatiotemporal distribution of cases, highlight current areas of interest (AOI) regions to conduct foci-specific targeted response, and extract supporting household and population data. GIS simulations were run to detect AOIs triggered throughout 2011 in each elimination province and conduct a sensitivity analysis to calculate the proportion of positive cases, households and population highlighted in AOI regions of a varying geographic radius.

**Results:**

A total of 183 confirmed cases were reported and mapped using the SDSS throughout 2011 and used to describe transmission within a target population of 90,354. Automatic AOI regions were also generated within each provincial SDSS identifying geographic areas to conduct response. 82.5% of confirmed cases were automatically geo-referenced and mapped at the household level, with 100% of remaining cases geo-referenced at a village level. Data from the AOI analysis indicated different stages of progress in each province, highlighting operational implications with regards to strategies for implementing surveillance-response in consideration of the spatiotemporal nature of cases as well as logistical and financial constraints of the respective programmes.

**Conclusions:**

Geospatial systems developed to guide Pacific Island malaria elimination demonstrate the application of a high resolution SDSS-based approach to support key elements of surveillance-response including understanding epidemiological variation within target areas, implementing appropriate foci-specific targeted response, and consideration of logistical constraints and costs.

## Background

As efforts to combat the global burden of malaria continue, there is a renewed focus on the need to strengthen surveillance throughout all phases of intensified malaria control and elimination [1-7]. This renewed emphasis on surveillance and its ultimate role in identifying and rapidly tackling remaining transmission reservoirs by adequate integrated response package is highlighted by the World Health Organization (WHO) Global Malaria Programme “T3: Test. Treat. Track” initiative and the publication of operational manuals to support surveillance-response approaches for malaria control and elimination [8-10].

As countries reduce transmission and progressively move from intensified control to pre-elimination, elimination and eventually the prevention of reintroduction, surveillance practices are required to evolve into a more focused intervention that incorporates passive, active and reactive case detection, and appropriate response measures [11,12]. For malaria elimination, where the goal is to halt localized transmission, the objective of surveillance systems is to rapidly detect, classify and attack all infection foci (both symptomatic and asymptomatic) to ensure all cases are treated before the occurrence of secondary cases and perpetuation of local transmission [10,13]. A detailed understanding of the local micro-epidemiological situation within target areas is essential to actively identify transmission foci and implement appropriate responses [14-17]. As transmission declines and locally acquired cases approach zero, sound vigilance also becomes essential to monitor outbreak and importation risk [18,19].

Whilst the integral role of surveillance in malaria elimination is recognized, significant challenges remain. Existing health systems often struggle to support the high level of cohesion, precision and responsiveness required to address the increased complexities of malaria elimination [4]. There is a call to explore novel technologies, approaches and decision-making tools to support the effective implementation and integration of surveillance-response into health systems managing intensified malaria control and elimination campaigns [1-4,11,20,21].

Since the global malaria eradication period of the 1950s–1960s, mapping and geographical reconnaissance (GR) has been a component of field-based vector control operations. However, limitations have included access to spatial data and the time consuming nature of manual hand-drawn cartographic, data collection and record keeping practices [11,22]. Surveillance-response approaches for malaria elimination is inherently geographic in its requirement to detect cases, locate transmission foci and target appropriate responses accordingly. Thus, mapping, including the use of contemporary geospatial technologies such as geographic information systems (GIS), global positioning systems (GPS), and high performance mobile computing and telecommunication devices, is likely to present considerable benefits. Whilst the potential of these geospatial resources is recognized, the development and practical implementation of modern, rapid fine-scale mapping tools at a resolution detailed enough to support surveillance-response requires further research [11].

Spatial decision support systems (SDSS) are computerized management systems designed to address complex geographic or spatial problems [23]. These systems are interactive and generally based around a GIS platform that integrates a database management system, graphical map interface, tabular reporting and expert knowledge of the user [23,24]. An SDSS provides a potential platform to process spatial information that is required to support surveillance-response decision making for malaria elimination [22].

Progressive malaria elimination campaigns are currently being pursued by the governments of Solomon Islands and Vanuatu, with support from the Australian Agency for International Development (AusAID) Pacific Malaria Initiative (PacMI), World Health Organization (WHO) and other partners. GIS-based SDSS approaches have been developed, validated and adopted by the malaria programmes in both countries to strengthen geographical reconnaissance (GR) [25] and to facilitate the implementation of vector control interventions including long-lasting insecticidal net (LLIN) distribution and focal indoor residual spraying (IRS) [22,26]. As these programmes progress, there is an increasing need to focus on the development, implementation and strengthening of effective surveillance-response to support elimination in the region [27,28]. This study focuses on the continued development of SDSS-based operational tools to support surveillance-response in both countries. Specific aims of this study were to develop a geospatial surveillance tool to automatically locate and map the distribution of reported confirmed malaria cases by household; and explore the effectiveness of an SDSS-based approach in supporting the rapid classification of identified active transmission foci to strategically guide targeted responses that can be applied within malaria elimination zones in the Asia-Pacific region and globally.

## Methods

### Study areas

**Figure 1:**
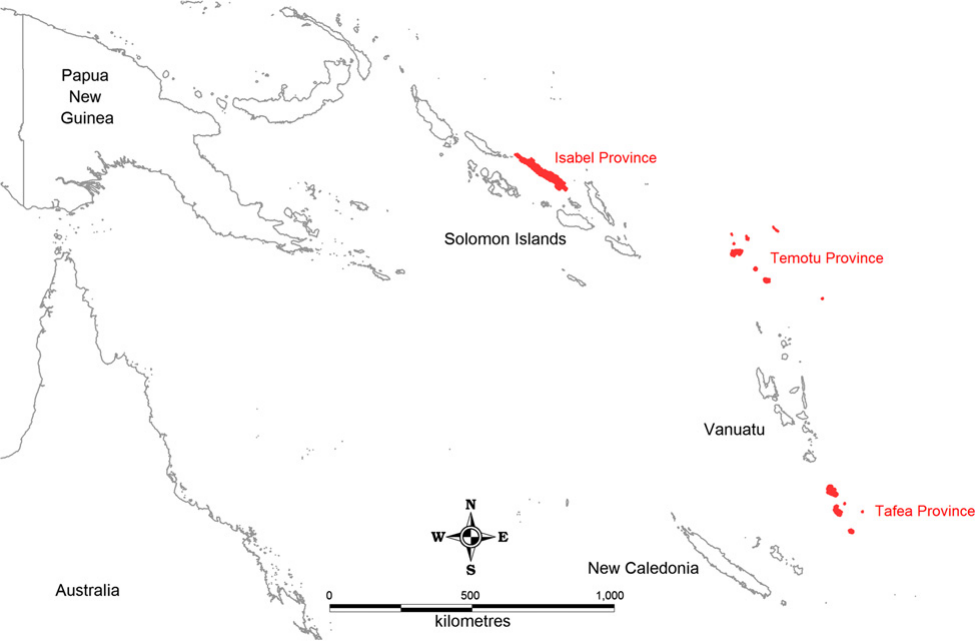
General location map of Solomon Islands and Vanuatu elimination provinces.

SDSS-based surveillance systems have been established in the malaria elimination provinces of Isabel and Temotu, Solomon Islands and in Tafea province, Vanuatu (Figure 1). Data on malaria cases collected in the three provinces throughout 2011 were used as the basis of this study. Approval for this study was provided by the Ministries of Health (MoH) in each country, who formally requested in-country technical support to assist in the development and implementation of a practical and easy-to-use SDSS-based malaria surveillance system to support progressive malaria elimination. Ethical approval was not sought during this study because the application and procedures developed are considered part of routine operational activities of the national malaria programmes in each country, with all data collected and managed as per confidentiality requirements of the Solomon Islands and Vanuatu Ministries of Health. Data collection activities and SDSS-based surveillance operations were conducted by provincial vector borne diseases control programme (VBDCP) surveillance, monitoring and evaluation (SM&E) officers as part of their routine activities, with technical assistance provided by the Pacific Malaria Initiative Support Centre (PacMISC) and WHO.

### Development of the spatial decision support system

Custom applications were built into the existing provincial SDSS used to support general topographic mapping, GR and vector control intervention management in the elimination provinces [25,26], using MapInfo Professional and MapBasic (Pitney Bowes Software Inc., NY, USA) GIS software. The existing system included enumerated and geo-referenced household data collected during detailed GR surveys conducted between 2008 and 2010 in all elimination provinces [25]. Specific components of the surveillance-response SDSS framework included the development of interactive GIS-based applications to map and define health facility catchment areas; automatically map confirmed malaria cases by household; automatically classify and map transmission foci based on the spatiotemporal distribution of cases and highlight “current areas of interest” to conduct re-active case detection and response activities. Table 1 provides a summary of the technical SDSS applications developed and their practical function for surveillance-response. A screenshot of the custom SDSS user interface is provided as Additional file 1.

**Table 1:** Summary of the technical components of the malaria elimination geospatial surveillance-response SDSS

**SDSS component**	**Technical function and application for surveillance response**
View / Map Positive Malaria Case Data	Automated synchronization between rapid case surveillance reporting database and GIS-based SDSS to geo-reference positive reported cases by household
	Positive case household mapping application to instantly view the spatial distribution of all geo-referenced positive reported cases via a GIS-based map
	Dialog window to enable SDSS-user to develop customized GIS maps of specific case data e.g. maps by species type, date range, and transmission mode to support epidemiological investigation
	Option to search, locate and access individual case data via a GIS-based map interface to support individual case investigation
Active Transmission Focus Mapping	GIS-based mapping application to automatically update, re-classify and map active transmission foci based on incoming reported positive cases; and epidemiological, entomological and environmental data
Current Area of Interest Focus Mapping	Interactive dialog window to enable SDSS-user to define the parameters to trigger current area of interests (AOI) i.e. The number of positive cases reported in a defined geographical radius of each other within a set time period
	Automated mapping application to generate current AOI maps to highlight current geographic locations to concentrate response actions based on user defined parameters
	Interactive map-based application to enable the SDSS-user to modify individual AOI regions automatically generated to adjust geographic areas based on local knowledge
	Automated GIS queries to extract specific data to support rapid response interventions within current AOI including general household and population summaries of all current AOIs; and historical case data, detailed household listings, and known larval site data by individual AOI
Health Facility Catchment Definition and Mapping	Interactive map-based application to draw and define health facility catchment boundaries via a graphical map interface
	Automated query to extract enumerated household lists and population data for individual catchment areas to support the geo-referencing at positive cases upon diagnosis and investigation
	Automated queries to produce general health catchment summaries by total population and households; and detailed individual health catchment summaries by village, population, age breakdown and households

Following consultation with national and provincial VBDCP partners in Solomon Islands and Vanuatu, forms were developed to communicate essential data on all confirmed malaria cases from health facilities for entry into the provincial-level SDSS. A simple database and data entry template was developed in Microsoft Access (Microsoft Corporation, Redmond, WA, USA). An automated link between the surveillance database and SDSS was developed to make reported case data available in the surveillance-response system in real time. A screenshot of the surveillance reporting data entry form template is also provided as Additional file 2.

### Development of Isabel Province surveillance-response conceptual framework

**Figure 2:**
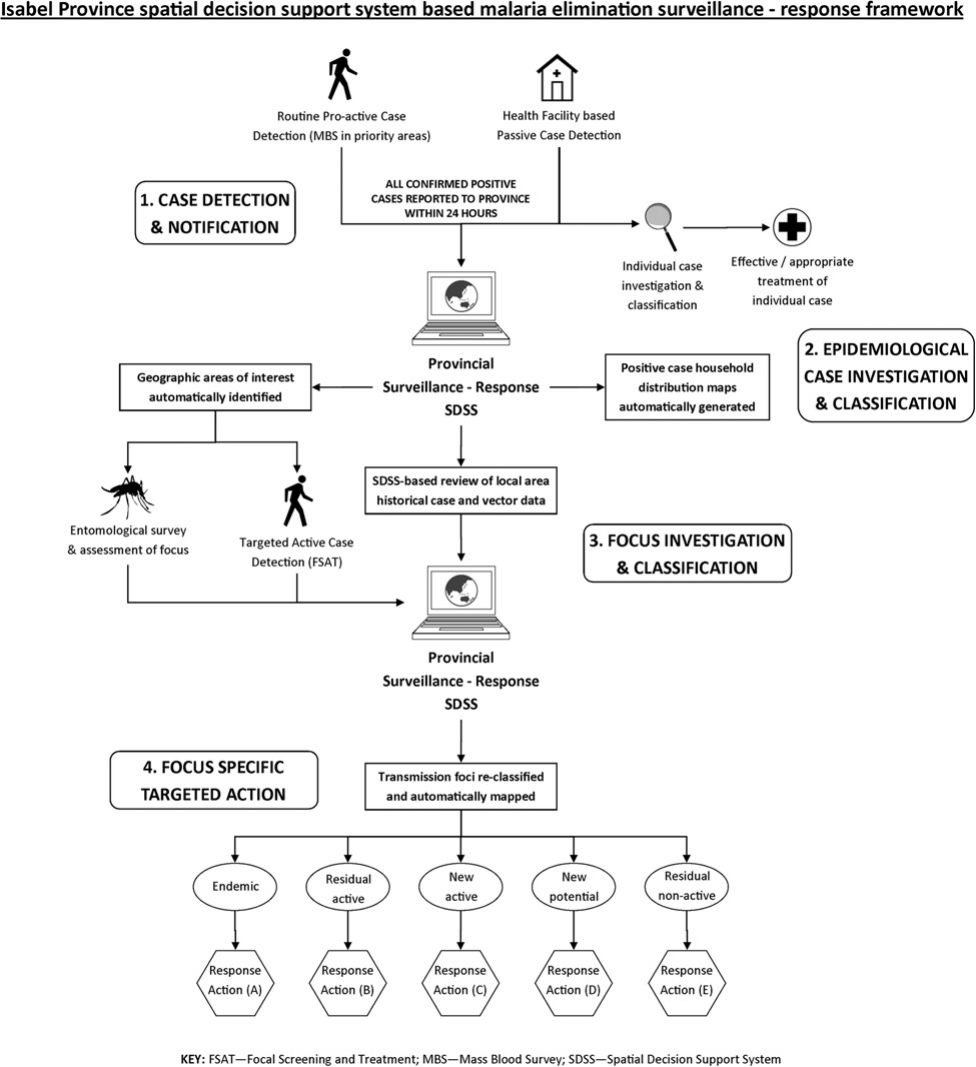
**Spatial decision support system based malaria elimination surveillance-response framework. **This schematic illustrates the SDSS-based framework adopted specifically for Isabel Province, Solomon Islands to guide surveillance-response operations. Key elements of the framework include: 1. Case detection and rapid notification via both passive and pro-active case detection methods; 2. Epidemiological case investigation and classification through automatic case mapping, and treatment and investigation of individual cases; 3. Focus investigation and classification via targeted re-active case detection and entomological assessment, historical case review, and automated GIS-based queries within the SDSS to classify and map transmission foci and generate response areas of interest (AOI); and 4. Focus specific targeted action based on identified AOI geographic areas and the classification of associated transmission foci (see Table 2).

Detailed consultations were held between national and provincial VBDCP personnel in Isabel province to develop a malaria elimination surveillance-response conceptual framework (Figure 2). Key elements discussed included the role of the SDSS in supporting rapid case reporting using passive and routine active case detection methods, the identification of current areas of interest (AOI) to conduct re-active focal screening and treatment (FSAT) based on reported cases and the automated classification and mapping of transmission foci to guide appropriate response measures in defined AOI.

To automate the identification of current AOI regions, GIS-based queries were developed and incorporated into the SDSS. AOI region parameters were based on interactive user-defined parameters designed to reflect the local spatiotemporal relationship of cases and transmission, the local vector flight range [29,30], and the operational capacity of the VBDCP office to conduct response in relation to current case-loads. Provincial SM&E officers were able to define the minimum number of positive cases reported within a defined geographical distance and date of notification of each other to trigger an AOI within the customized geospatial surveillance-response SDSS. As cases were uploaded into the SDSS, buffer regions automatically mapped the geographic areas meeting the minimum AOI requirements to highlight areas requiring a follow-up response. Associated household, population and transmission data of each AOI were also automatically generated to support reactive response measures in these areas. Initial AOI parameters in Isabel were set at two or more cases within 2 km, reported within the last 90 days.

Following consultations held in Isabel, automated GIS queries were integrated into the SDSS to automatically classify and map active (and residual non-active) transmission foci based on localized geographic, epidemiological, and entomological factors. Table 2 provides a summary of the parameters used to automatically classify and map these transmission foci in the SDSS and the associated response interventions selected by the VBDCP.

**Table 2:** Active and residual non-active transmission foci classification parameters and nominated response interventions adopted in Isabel province surveillance-response SDSS

**Focus type**	**Characteristics**	**SDSS classification parameters**	**Nominated response interventions**
Residual active	- Transmission occurring in an area of ongoing transmission	All areas within a 3km geographical range of a positive case in an area that has had one or more additional positive recorded case within the last 3 months	Focal screening and treatment
			Larval source management
	- Effectively controlled with major reductions recorded after interventions		Updated geographical reconnaissance
			Community Awareness
New active	- Transmission occurring in area that has had transmission for less than 2 years or has never had local transmission - 1^st ^Degree: Only introduced cases present; 2^nd ^Degree: Secondary and indigenous cases present	All areas within a 3km geographical range of a positive case in an area of known transmission but has not had an additional reported case within 3 months	Focal screening and treatment
			Focal indoor residual spraying Long lasting insecticidal net assessment and re-distribution as required Larval source management Updated geographical reconnaissance Community Awareness
New potential	- Isolated imported, induced or relapse cases occurring only	All areas within a 3km geographical range of an imported positive case in a known receptive area that has not had transmission for a period of 2 years	Focal screening and treatment
			Focal indoor residual spraying
	- Receptive area with no transmission for at least 2 years		Long lasting insecticidal net assessment and re-distribution as required
			Larval source management
			Updated geographical reconnaissance
			Community Awareness
Residual Non-active	- History of local transmission however not within the last 2 years - Relapses or delayed primary infections with P. vivax or treatment failure of infection before transmission	All areas within a 3km geographical range of a relapsed case in a known receptive area that has not had transmission for a period of 2 years	Focal screening and treatment
			Direct observed treatment and case follow-up Updated geographical reconnaissance Community Awareness

### Rapid case reporting

Protocols developed for Isabel, Temotu and Tafea provinces specified that all positive cases, confirmed by Giemsa-stained blood smear examination or ICT Malaria Combo Cassette Test (ICT diagnostics) rapid diagnostic test (RDT), were to be reported to the provincial VBDCP offices within 24 hours by mobile phone (if available) or radio communication. Case data were updated into surveillance-response SDSS by provincial SM&E officers using the surveillance reporting database form. The spatial and temporal distributions of positive malaria cases were then viewed and monitored by malaria surveillance staff using the SDSS map interface. Case distribution maps and tabular summaries were developed and reported to the respective national malaria offices as part of routine monthly reporting procedures.

To support the rapid reporting of positive cases, health facility catchment areas were mapped in the SDSS during planning sessions by health facility staff and provincial VBDCP SM&E officers, using the existing geo-referenced GR household data. Hardcopy household location maps and associated lists showing household number, family name, village and household demographics were then issued to all health facilities. These data were used by health facility officers during diagnosis and case investigation to report the suspected site of transmission to the provincial VBDCP office. Hardcopy and digital household location maps and associated lists were also stored at the provincial level and could be accessed by provincial VBDCP staff in the customized SDSS.

### Training

Introductory 2-day training sessions were held in Isabel, Temotu and Tafea province respectively, covering the basic operation of the customized SDSS. Standard operating procedures (SOPs) were also developed to guide reporting procedures and support technical operations. Briefings with health facility officers were conducted on rapid case reporting procedures. Support was also provided to provincial surveillance officers through regular consultation, both remotely and during routine provincial visits.

### Evaluation and validation of the spatial decision support system

Confirmed positive case data reported throughout 2011 were used to evaluate the geospatial surveillance-response SDSS. Local and imported case data summaries, case household distribution maps, AOI maps and associated data summaries were automatically produced for Isabel, Temotu and Tafea provinces using the customized SDSS. Local cases were defined as infections that were classified as having been acquired within each respective elimination province, with imported cases classified as an infection acquired outside of the individual respective elimination province.

Simulations based on varying AOI criteria were also run to calculate the number of current AOIs triggered throughout 2011 in Isabel, Temotu and Tafea and the proportion of positive cases, households and population highlighted in the identified areas. A sensitivity analysis for the AOI criteria was conducted using a simulation approach, with the criteria varied as follows: (i) two or more cases within a 1 km radius of each other within 90 days (2c1km90d); (ii) two or more cases within a 2 km radius of each other within 90 days (2c2km90d); and (iii) two or more cases within a 3 km radius of each other within 90 days (2c3km90d).

The time taken to report and update cases into the SDSS from the date of diagnosis was recorded in Isabel province to evaluate the timeliness of case reporting. The proportion of confirmed positive cases successfully geo-referenced by households were also examined in the three elimination provinces to assess the effectiveness of the geospatial surveillance-response SDSS.

## Results

### Case detection and mapping

A total of 183 confirmed positive malaria cases were reported and recorded in the three provincial surveillance-response SDSS throughout 2011. Of these confirmed cases, 26 were reported in Isabel Province and 141 in Temotu Province, Solomon Islands; and 16 in Tafea Province, Vanuatu. Suspected local transmission accounted for 61.6%, 95.0% and 68.8% of all reported cases in Isabel, Temotu and Tafea respectively. *Plasmodium vivax* was the dominant species reported in all elimination provinces accounting for 76.9%, 92.2% and 68.8% in Isabel, Temotu and Tafea respectively. Table 3 provides a breakdown of positive cases by species type and suspected mode of transmission by province in 2011.

**Table 3:** Breakdown of 2011 case species type and suspected mode of transmission

**Elimination area**	**P. vivax**		**P. falciparum**		**Mixed**		**Total**		**Total Cases**
	**Local**	**Imported**^**1**^	**Local**	**Imported**^**1**^	**Local**	**Imported**^**1**^	**Local**	**Imported**^**1**^	
Isabel	11	9	4	1	1	0	16	10	26
Temotu	125	5	8	2	1	0	134	7	141
Tafea	7	4	4	1	0	0	11	5	16
Total	143	18	16	4	2	0	161	22	183

^1 ^Imported case is defined as an infection classified as being acquired outside of the respective elimination province.

**Figure 3:**
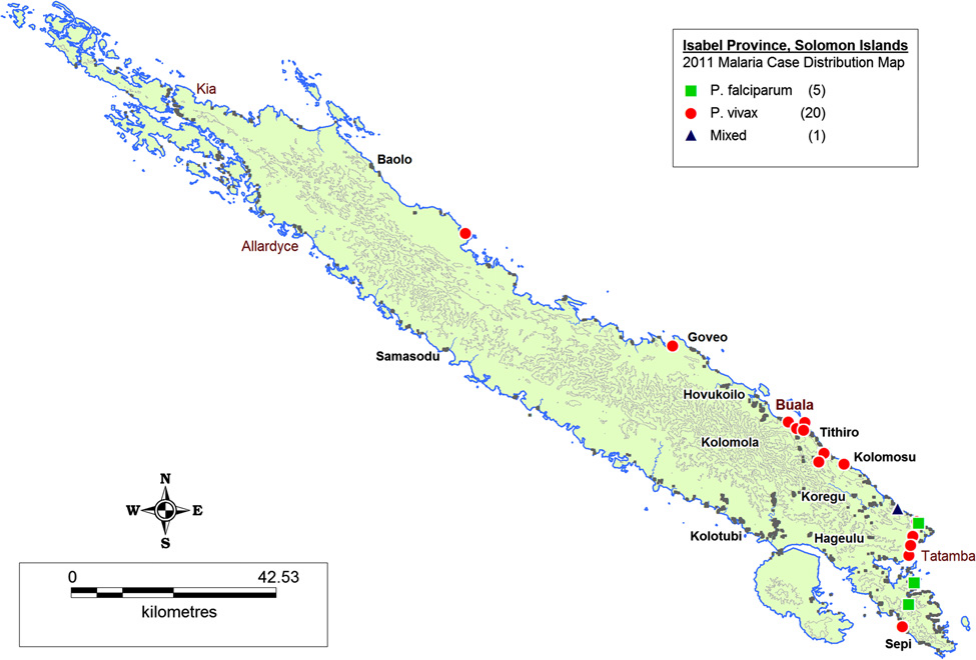
Malaria case distribution map, Isabel Province, Solomon Islands, 2011.

**Figure 4:**
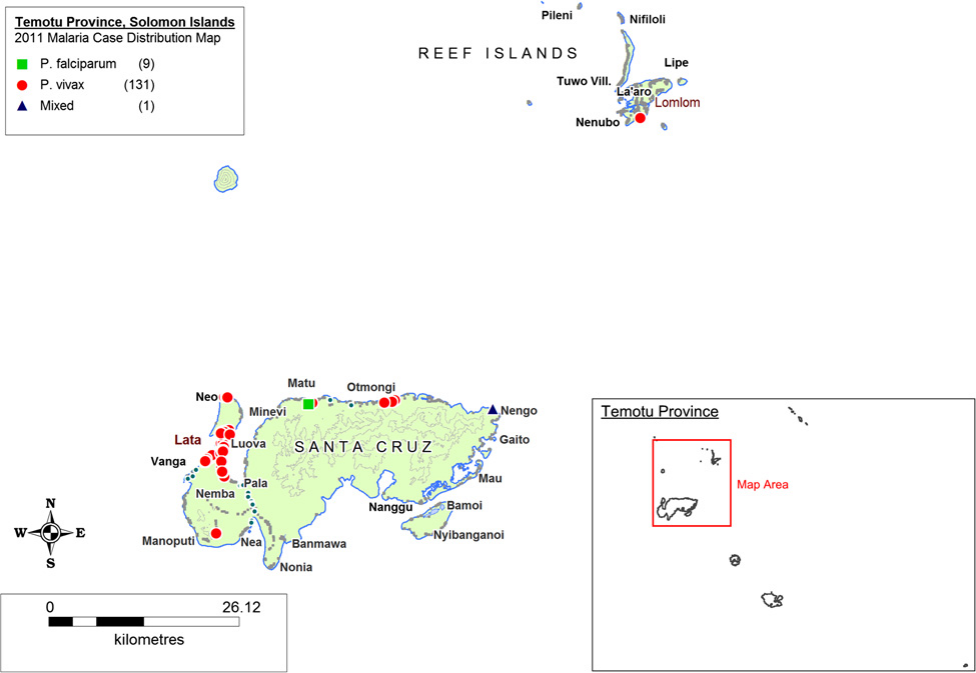
Malaria case distribution map, Temotu Province, Solomon Islands, 2011.

**Figure 5:**
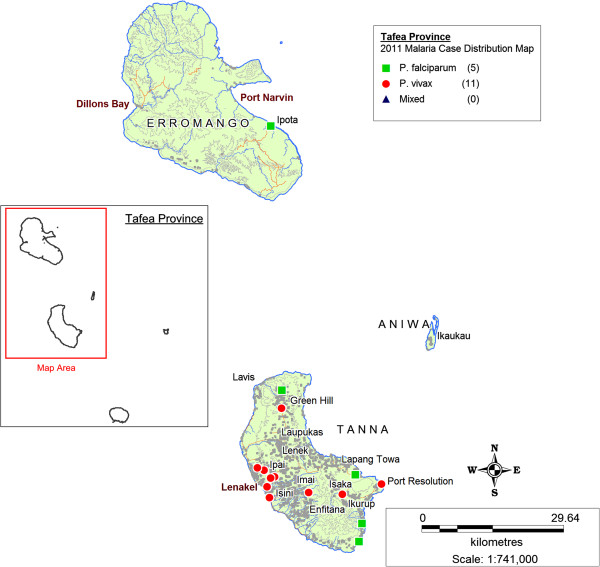
Malaria case distribution map, Tafea Province, Vanuatu, 2011.

Figures 3, 4 and 5 illustrate maps of cases generated for Isabel, Temotu and Tafea by species in 2011. There were no technical or operational issues reported in any elimination province that impeded the day-to-day generation of SDSS-based case distribution maps by the provincial surveillance teams.

Transmission in Isabel Province was concentrated in the residual active areas of the south-eastern populated communities of the Tatamba region on the main island as well as the provincial centre of Buala (Figure 3). Isolated imported cases were also reported in logging camps along the north-western coastline of Isabel, detected both passively and as part of routine active case detection (ACD). Transmission in Temotu Province was predominately concentrated in the provincial capital and main entry port of Lata on the main island of Santa Cruz, with additional reported cases reported in active transmission areas of the north coast of Santa Cruz (Figure 4). In Tafea, transmission was somewhat sporadic, with the majority of cases reported on the main island of Tanna, particularly around the provincial capital of Lenakel (Figure 5).

**Figure 6:**
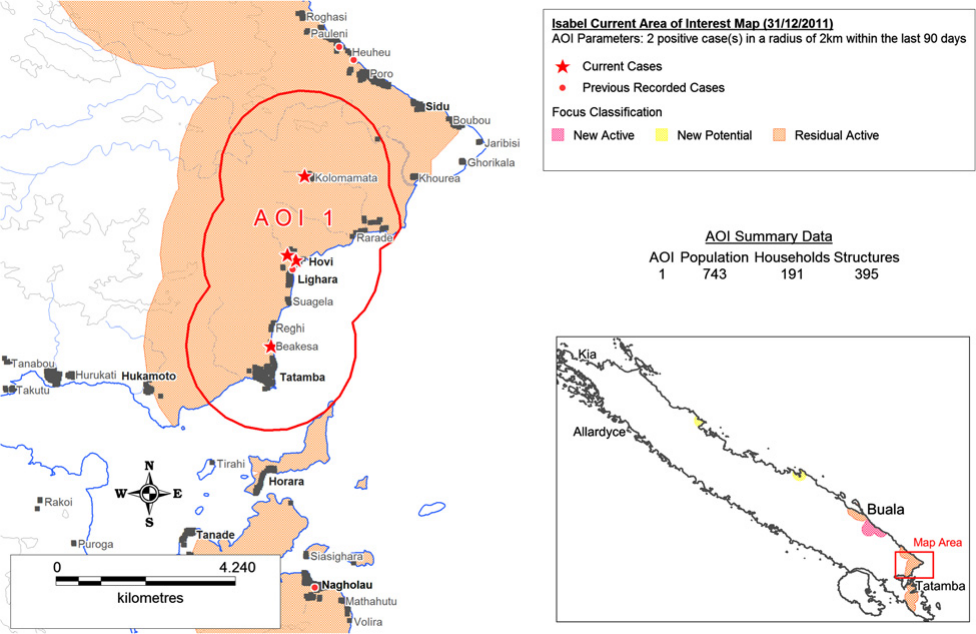
**Current Area of Interest (AOI) map, Isabel Province, Solomon Islands, 31/12/2012.** This map illustrates a “Current AOI map” generated using the Isabel Province surveillance-response SDSS for 31^st ^December, 2011. The AOI area illustrated was automatically generated in the SDSS based on the AOI defined parameters of two or more malaria cases detected within a two kilometre radius of each other within the last 90 days of the current date (31/12/2012). Key elements of the automated map include: (i) the designation of the geographic AOI area to conduct response; (ii) the type of transmission focus the AOI is located within to guide the selection of nominated response interventions; (iii) the illustration and generation of household and population data within the AOI to support rapid resource allocation, costing, and field implementation of nominated interventions.

Figure 6 illustrates an AOI map generated in Isabel Province on 31^st^ December 2011 based on the default AOI parameters of 2 or more positive cases reported within a geographic radius of 2 km in the last 90 days.

### Comparison of “Area of Interest” parameters

**Figure 7:**
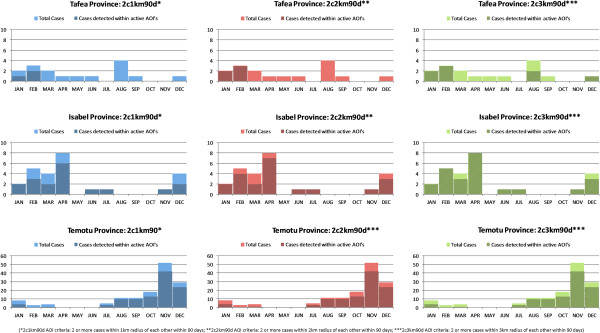
**Proportion of positive case per month against detected AOI regions of varying geographic radius. **Figure illustrates the temporal distribution of cases in each elimination province in relation to area of interests (AOI) of varying geographic radius 1 km, 2 km, 3 km. Figure shows the total number of cases reported by month and the proportion of those reported cases located within an active AOI geographical region based on the set parameters defined within the SDSS.

Table 4 provides a detailed summary of the AOI simulation data for Isabel, Temotu and Tafea provinces comparing variations between geographic radius parameter between 1 km, 2 km and 3 km. Figure 7 illustrates the total proportion of positive reported cases per month against detected AOI regions for each AOI simulation. A relatively steady increase in the proportion of cases detected in Isabel was observed with the 1 km, 2 km, and 3 km AOI criteria, with 69.2%, 80.8%, and 92.3% respectively. In Temotu, no difference was recorded in the proportion of cases (76.0%) detected based on the 1 km, 2 km, and 3 km AOI criteria. Whilst no difference in the proportion of cases was recorded, an increase of 1298 population (6.0% of the total provincial population) and 311 households (6.0% of total provincial households) was identified between the 1 km and 3 km AOI regions. In Tafea, a relatively small proportion of cases (18.8%) were detected based on the 1 km geographic radius parameter, increasing to only 50% of total cases detected when the AOI radius was increased to 3 km. Based on the 1 km and 2 km parameters, only January and February recorded AOI regions in Tafea during 2011.

**Table 4:** Proportion of cases detected and associated population and households located within SDSS generated areas of interest (AOI) of varying radius

		**Isabel**	**Temotu**	**Tafea**	**Total**
**Total**	**Cases**	26	141	16	183
	**Pop^**	30167	21552	38635	90354
	**HHs^^**	6410	5221	8850	20481
2c1km90d*	**Cases**	69.23%	75.89%	18.75%	69.95%
		[18]	[107]	[3]	[128]
	**Pop**	11.96%	23.96%	5.12%	11.90%
		[3608]	[5163]	[1980]	[10751]
	**HHs**	10.66%	23.64%	5.14%	11.58%
		[683]	[1234]	[455]	[2372]
2c2km90d**	**Cases**	80.77%	75.89%	31.25%	72.13%
		[21]	[107]	[5]	[132]
	**Pop**	23.34%	26.88%	17.90%	21.86%
		[7040]	[5793]	[6917]	[19750]
	**HHs**	22.40%	26.49%	17.67%	21.40%
		[1436]	[1383]	[1564]	[4383]
2c3km90d***	**Cases**	92.31%	75.89%	50.00%	75.41%
		[24]	[107]	[8]	[138]
	**Pop**	30.47%	29.98%	31.76%	30.91%
		[9193]	[6461]	[12270]	[27924]
	**HHs**	29.75%	29.59%	31.94%	30.66%
		[1907]	[1545]	[2827]	[6279]

(^Pop: Population; ^^HHs: Households; *2c1km90d AOI criteria: 2 or more cases within 1 km radius of each other within 90 days; **2c2km90d AOI criteria: 2 or more cases within 2 km radius of each other within 90 days; ***2c3km90d AOI criteria: 2 or more cases within 3 km radius of each other within 90 days).

### Timeliness of reporting of cases

Table 5 provides a breakdown of the recorded times taken for the Isabel provincial office to receive notification of cases from the health facility and updated into the surveillance-response SDSS in 2011. Of the total recorded cases, 46.2% of cases were received within 24 hours, as required in the rapid reporting protocol.

**Table 5:** Time taken to report cases from health facility to Isabel provincial office during 2011

**Time taken for provincial office to receive case notification**	**Total cases**	**Proportion of cases**
Reported within 24 hours	12	46.15%
Reported within 72 hours	0	0%
Reported within 1 week	9	34.62%
Reported within 1 month	4	15.38%
Reported after 1 month	1	3.85%

### Geo-referencing of reported positive cases by household

A high proportion of positive cases were successfully geo-referenced by household in 2011 with 100% of positive cases in Isabel, 78.0% in Temotu, and 93.8% in Tafea (Table 6). Common reasons that cases were not geo-referenced by household included: confusion among health facility workers about how to assign household numbers to visitors, the construction of new houses without a geo-referenced household number, and a lack of understanding on the importance of reporting household numbers as part of the elimination surveillance-response system. Of the cases that were not successfully geo-referenced by household, 100% were successfully geo-referenced at the village level by provincial surveillance officers.

**Table 6:** Proportion of 2011 reported positive malaria cases successfully geo-located at the household level

**Elimination area**	**Total cases**	**Cases geo-located**	**Percent (%) geo-located**
Isabel	26	26	100.00
Temotu	141	110	78.01
Tafea	16	15	93.75
Total	183	151	82.51

## Discussion

This paper demonstrates the use of geospatial technology and processes as part of integrated surveillance-response approaches for malaria elimination in Solomon Islands and Vanuatu. In all three elimination areas, the provincial VBDCP office serves as the focal point for the coordination and implementation of malaria elimination activities. Development of the SDSS-based systems focused on providing a practical, user friendly operational tool at the provincial level to support detailed surveillance-response, based on routinely collected programme data and existing health systems currently in place.

Logistical constraints associated with poor transport and communications infrastructure, limited resources and isolated communities present significant operational challenges for the effective delivery of essential services in Pacific Island settings where malaria elimination is currently being pursued. A need for effective operational tools to accurately identify transmission foci, and support the efficient targeting of response interventions and timely allocation of associated resources to designated geographic locations is crucial to ensure local transmission can be effectively halted. The ability to automatically map both passively and actively detected cases in the SDSS provides an effective mechanism to visualize the distribution and pattern of malaria transmission in these elimination settings at high resolution and in relative real-time (i.e. as soon as cases are reported). As Figure 6 indicates, a SDSS-based framework also allows for the application of powerful GIS capabilities to query the spatiotemporal characteristics of current and historical case data in relation to local geographic settings. Such a framework enables programme managers to locate individual positive cases at a detailed household level; identify, select and map priority AOI regions; guide the selection of appropriate focus-specific response interventions; and extract detailed supplementary data (such as population summaries and household listings). Access to such detailed data supports swift and effective decision-making in peripheral and remote areas including the rapid preparation of budgets, allocation of required resources and mobilization of personnel to support the implementation of interventions within identified transmission foci.

The data presented from 2011 indicate different stages of progress in each of the elimination provinces. With the limited resources, available finances and logistical constraints characterising these areas, the total number of reported cases occurring in each elimination zone has operational implications with regard to the current nature of surveillance-response approaches that each programme can effectively carry out. In Temotu province, where total reported cases were highest, targeted responses based on individual cases is not yet operationally feasible. Whilst transmission is comparatively high in Temotu, the positive case household map (Figure 4) illustrates the largely clustered and heterogeneous nature of transmission in the province. This clustering is also indicated in the AOI simulation data (Table 4, Figure 7). In the context of managing surveillance-response, these data suggest that re-active case detection and response interventions could initially be targeted to a smaller geographic radius (e.g. 1 km) in these transmission foci, utilising less resources and finances to seek out and treat additional asymptomatic cases and efficiently attack transmission. In contrast, in Tafea province, where total positive cases reported were comparatively lower, the spatiotemporal distribution of cases was more sporadic and dispersed, with only a small proportion of cases detected during the AOI simulations throughout 2011. These data suggest the readiness and need to adjust AOI parameters in Tafea to trigger response interventions based on single cases to maximize the operational impact of the implemented surveillance-response interventions to support the elimination of remaining parasite reservoirs and prevent re-introduction of local transmission.

Complexities associated with the heterogeneous nature of malaria transmission (both spatial and temporal) and additional factors such as the risk of re-introduction into non-active receptive areas through imported cases, present management challenges for elimination that require a multifaceted approach to decision-making and response. An advantage of the SDSS-based surveillance-response system presented in this paper is its ability to provide surveillance officers with a tool to visualize the variation in current transmission across the entire elimination zone and adjust AOI regions according to the local situation to rapidly extract detailed information, including costs, to guide appropriate integrated response packages in various transmission settings.

Health systems challenges in Solomon Islands and Vanuatu currently pose constraints to the effectiveness of the SDSS based surveillance-response systems in the three selected elimination provinces. As the SDSS system relies upon on effective passive case detection (illustrated in Figure 2), the performance of this system is dependent upon key health system components, particularly at the health facility and community levels. These challenges include the need to strengthen community engagement and participatory surveillance to encourage early treatment seeking behaviour and community level vigilance [27,28]; accurate diagnostics; timely and effective reporting of cases from the health facility to provincial level via suitable communication channels; and robust pro-active case detection and increased vigilance in high risk priority areas such as known populations of high mobility (e.g. logging and mining camps) and common entry points (e.g. sea and air ports).

Previous malariometric surveys conducted in the elimination provinces have revealed a high burden of asymptomatic malaria infections of low parasite density [27,31,32]. Use of sensitive field-based molecular methods will likely be required to effectively detect these low level infections [31]. As these operations are implemented, reported cases will need to be integrated into the SDSS-based surveillance-response framework to track progress and continue to provide appropriate response interventions.

The effectiveness of the SDSS surveillance-response system is also highly dependent upon the timely and accurate reporting of cases at the health facility level. As reflected in Table 5, significant challenges remain to ensure cases are immediately reported to the provincial level to update the SDSS surveillance-response system. Current plans to utilize digital mobile communication technologies to support rapid case reporting from the health facility in the elimination provinces are underway. The capacity of health facilities to geo-reference data and correctly classify cases also impacts on the ability of the SDSS to automatically map the distribution of cases and accurately classify transmission foci, particularly as the number of cases detected heads towards zero. Policies to conduct updated and re-active GR mapping as required and standard procedures to support case investigation and classification have been incorporated into the surveillance-response framework to mitigate these particular operational challenges. In conjunction with these technological and procedural mechanisms, further emphasis on supporting health worker performance and reinforcing the importance of the immediate and accurate reporting of geo-referenced positive cases at the health facility level as part of an effective surveillance-response framework for malaria elimination is essential.

Currently all case detection in the elimination provinces of Solomon Islands and Vanuatu occurs within the public sector. In areas where both public and private sectors operate concurrently, additional effort would be required to capture and include in the surveillance-response framework malaria cases detected in patients seeking health care through the private sector.

Adequate response capacity is essential to any effective surveillance package [13]. Whilst the SDSS surveillance-response system provides a framework to identify priority areas for response and guide the selection of appropriate integrated interventions, further work is still required in all three provinces with regard to the practical implementation and reporting of targeted responses interventions, including reactive case detection, in areas identified by the SDSS-based surveillance-response system. As this paper largely focuses on surveillance and the identification of target response areas, the effective identification and monitoring of appropriate foci-specific interventions and adequate response times remain future research questions.

## Conclusion

This study has illustrated the application of SDSS technology to support high-resolution surveillance-response for malaria elimination in remote Pacific Island settings. This study has shown how an SDSS based approach to surveillance-response can utilize GIS queries to support the identification and mapping of malaria cases at a detailed household level, visualize and classify active transmission foci, detect priority geographic areas to conduct follow-up activities, and automatically extract detailed data to support the rapid mobilization of appropriate response interventions. When integrated into existing health systems, an SDSS framework provides programme managers with an effective and flexible operational tool to actively understand micro and meso-epidemiological variations within an elimination area and respond accordingly. SDSS-based geospatial surveillance-response systems currently remain in operation in all three elimination provinces in the Solomon Islands and Vanuatu. Further refinement and validation of these systems and their extension to include a costing assessment of the surveillance system and associated response packages are currently continuing, as well as additional applications being developed to support intensified malaria control and broader disease surveillance within the region.

## Competing interests

The authors declare that they have no competing interests.

## Authors’ contributions

The study was conceived and designed by GK, with support from ACAC, MT, AV, LV, AB & GT. Field training was conducted by GK, JS, EH & WD. Field research operations were coordinated by EH, WD, WB, JN, GK & JS. ACAC provided guidance on scientific aspects of the study and provided detailed commentary on manuscript drafts. Additional scientific and technical guidance was provided by MT, AV, LV, KP & HB. Manuscript drafting was carried out by GK with support from all authors. All authors read and approved the final manuscript.

## Supplementary Material

Additional file 1 Screenshot of the Isabel Province surveillance-response spatial decision support system user interface. Click here for file

Additional file 2 Screenshot of the Temotu Province rapid case surveillance reporting digital data entry form template. Click here for file
