# Supplementary figures and images for: A high-resolution geospatial surveillance-response system for malaria elimination in Solomon Islands and Vanuatu
Source: Malar J. 2013 Mar 21;12:108. doi: 10.1186/1475-2875-12-108 (PMC3618239; doi:10.1186/1475-2875-12-108)

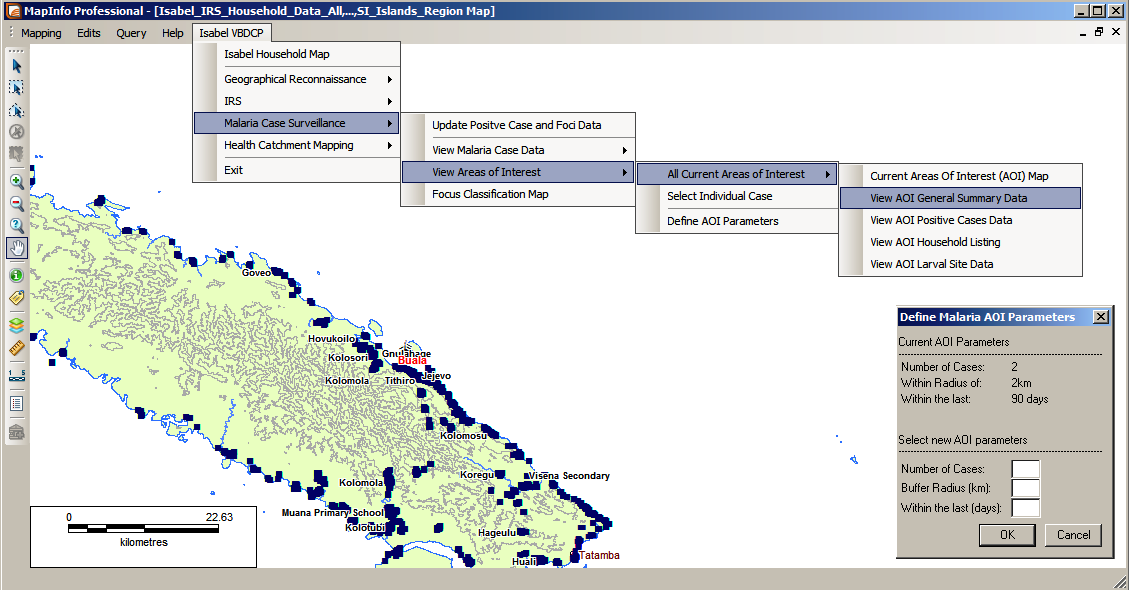

Supplement: Additional file 1 — Screenshot of the Isabel Province surveillance-response spatial decision support system user interface. [file 1475-2875-12-108-S1.tiff]

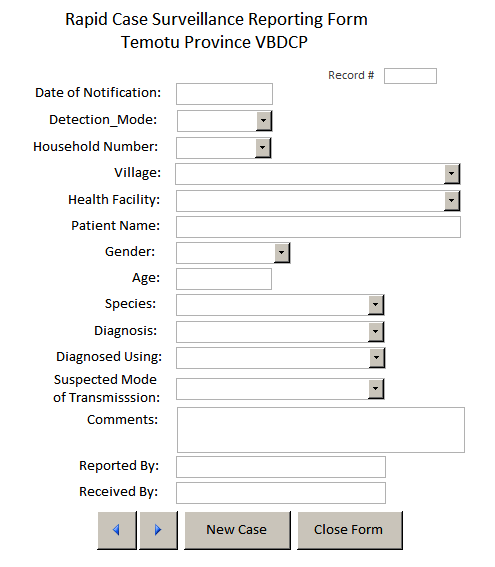

Supplement: Additional file 2 — Screenshot of the Temotu Province rapid case surveillance reporting digital data entry form template. [file 1475-2875-12-108-S2.tiff]
